# Impact of androgen deprivation therapy on cognitive function in men with prostate cancer

**DOI:** 10.1002/bco2.319

**Published:** 2023-12-11

**Authors:** Yutaka Yamamoto, Yasunori Akashi, Keisuke Kiba, Akihide Hirayama, Hirotsugu Uemura

**Affiliations:** ^1^ Department of Urology Kindai University Nara Hospital Ikoma Japan; ^2^ Department of Urology Kindai University Faculty of Medicine Osakasayama Japan

**Keywords:** androgen deprivation therapy, cognitive function, prostate cancer

**Abbreviations**
ADT: androgen deprivation therapy
HCs: healthy controls
IMRT: intensity‐modulated radiotherapy
LH–RH: luteinizing hormone–releasing hormone
MMSE: mini‐mental state examination
PC: prostate cancer
SD: standard deviation

## 1 INTRODUCTION

While the benefits of androgen deprivation therapy (ADT) have been well established for prostate cancer (PC) patients, it is also known to cause various adverse events as a consequence of testosterone deficiency.^1^ Recently, there has been growing interest in the association between ADT and cognitive function, but no consensus has yet been reached.^2^ Androgen receptors are highly expressed in the prefrontal cortex and hippocampus in the brain cortices and these regions are considered to responsible for memory and higher order cognitive functions.^3^ ADT for suppressing androgen release may lead to cause decreased activity in these regions, resulting in cognitive decline.^4^ Given that both the prevalence of cognitive decline and the number of ADT users increase with age,^5^ it is important to articulate the impact of ADT on cognitive function. The aim of this study was to evaluate the impact of ADT on cognitive function over a 36‐month follow‐up, including changes in serum testosterone levels.

The following two cohorts were enrolled in this study between July 2017 and April 2019 from Kindai University Nara Hospital in Nara, Japan: men with newly diagnosed PC who received ADT and healthy controls (HCs) without PC. Of those, 43 with PC and 34 HCs were evaluated in this analysis. All men with PC received a luteinizing hormone–releasing hormone (LH–RH) agonist. The addition of anti‐androgen was administered at the discretion of the attending physician. The vast majority of localized or locally advanced PC received intensity‐modulated radiotherapy (IMRT) in combination with ADT. Cognitive function was assessed using the mini‐mental state examination (MMSE),^6^ and a score of less than 24 out of 30 were excluded from this study. MMSE and blood samples for total testosterone were collected at baseline, 6 months, 12 months and 36 months. We also examined the percentage of MMSE domains in each cohort that had a change of 1 standard deviation (SD) (improved or worsened) from baseline to 36 months. Changes in cognitive function and serum testosterone levels between baseline and each follow‐up visit were compared by linear mixed effect models. An analysis of changes in the percentage of MMSE domains in each cohort was performed using an unadjusted chi‐square test. This study was approved by the Institutional Review Board of our hospital.

**FIGURE 1:**
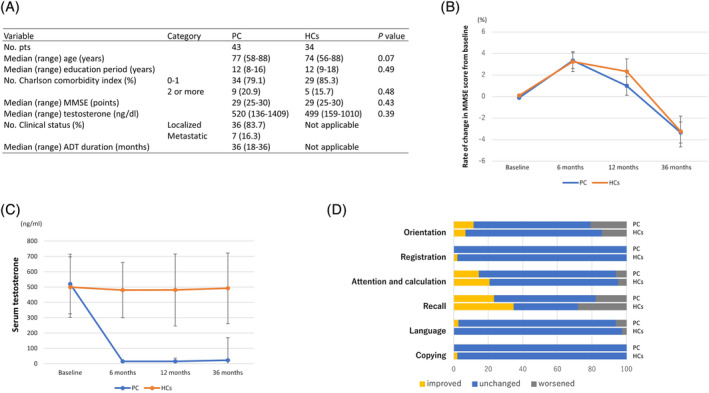
(A) Baseline characteristics of participants. ADT, androgen deprivation therapy; HCs, healthy controls; MMSE, mini‐mental state examination; PC, prostate cancer. Baseline characteristics were described using medians for continuous variables and percentages for categorical variables. *p*‐values were calculated using Student's *t*‐test for continuous variables, and chi‐square test for categorical variables. (B) Rate of change in cognitive function assessed using MMSE during 36 months. No significant difference between the two cohorts was found in cognitive function at each observation point (*p =* 0.38). Error bars represent standard deviation (SD). (C) Changes in serum testosterone levels during 36 months. A significant decrease in serum testosterone levels was observed in PC but not in HCs at each observation point (*p*‐value < 0.01). Error bars represent SD. (D) Percentage of individuals shown by cohort demonstrating improved (shown in yellow), not changed (shown in blue) or worsened (shown in grey) in each domain from baseline to 36 months. Results showed no statistically significant differences between the two cohorts in each domain.

Figure 1A shows the baseline characteristics. Median age, baseline MMSE score, Charlson comorbidity index, education level and serum testosterone levels were well matched between the two cohorts. The median duration of ADT was 36 (range 18–36) months. The rate of change in MMSE score from baseline is shown in Figure 1B. From baseline to 36 months, there was no significant difference in cognitive function between the two cohorts at each observation point (*p =* 0.38). We also stratified the PC cohort based on the use of anti‐androgens and examined the correlation with MMSE change, but no significant differences were observed (*p =* 0.54 [other data not shown]). Figure 1C shows the changes in serum testosterone levels. A significant decrease in serum testosterone levels below the castration level (<50 ng/dL) was observed in PC but not in HCs, with a significant difference at 6, 12, and 36 months, respectively (*p* < 0.01). An analysis examining the percentage of subjects per cohort in each domain of cognitive function from baseline to 36 months is illustrated in Figure 1D. Results showed no statistically significant differences between the two cohorts in each domain.

In a 36‐month follow‐up, we found that ADT was not associated with cognitive decline even when serum testosterone levels were maintained below the castration level. To the best of our knowledge, only Alibhai et al. reported the changes in cognitive function in men receiving ADT for over a year.^7^ They concluded that use of ADT for up to 36 months was not associated with cognitive decline, which is consistent with our results. Given that the majority of men receiving ADT do so for several years in real‐world clinical practices, our findings could provide valuable information to assess the risks and benefits of ADT.

Our study has several strengths, including longitudinal assessments over 3 years and detailed serum testosterone assessments. However, there are several limitations, including the relatively small sample size and the absence of PC patients who have not received ADT in the control group. A significant limitation of our study is that we assessed it with a single neuropsychological test. Further studies with multiple neuropsychological assessments are warranted.

## AUTHOR CONTRIBUTIONS

Yutaka Yamamoto and Akihide Hirayama proposed and designed the experiments; Yutaka Yamamoto, Yasunori Akashi and Keisuke Kiba performed the experiments; Yutaka Yamamoto and Keisuke Kiba analysed the data; Yutaka Yamamoto wrote the manuscript; Akihide Hirayama and Hirotsugu Uemura supervised this study. All authors approved the final manuscript.

## CONFLICT OF INTEREST STATEMENT

All authors declare that there are no conflicts of interest.
